# Centering PrEP: Utilizing ADAPT-ITT to inform group PrEP care for sex workers in Chicago

**DOI:** 10.1186/s12889-023-17508-4

**Published:** 2024-01-02

**Authors:** Randi Beth Singer, Janelli Barrow, Amy K. Johnson, Jessica Zemlak, Natasha Crooks, Sarah Abboud, Douglas Bruce, Noel Green, Jahari Stamps, Jennifer Neely, Susan G. Sherman, Crystal L. Patil, Alicia K. Matthews

**Affiliations:** 1 College of Nursing, University of Illinois Chicago (UIC), Chicago, IL USA; 2 Lurie Children’s Hospital, Chicago, IL USA; 3 College of Nursing, Marquette University, Milwaukee, WI USA; 4 Chicago Center for HIV Elimination, Chicago, IL USA; 5 Southside Health Advocacy Resource Partnership, Chicago, IL USA; 6 Northwestern Memorial Hospital, Chicago, IL USA; 7 Bloomberg School of Public Health, Johns Hopkins University, Baltimore, MD USA; 8 College of Science and Health, DePaul University, Chicago, IL USA; 9 School of Nursing, Columbia University, New York, NY USA; 10 School of Nursing, University of Michigan, Ann Arbor, USA

**Keywords:** HIV, sex work, ADAPT-ITT, group care, PrEP, community empoerment

## Abstract

**Background:**

Sex workers, those who trade sex for monetary or nonmonetary items, experience high rates of HIV transmission but have not been adequately included in HIV prevention and Pre-Exposure Prophylaxis (PrEP) adherence program development research. Community-empowered (C.E.) approaches have been the most successful at reducing HIV transmission among sex workers. Centering Healthcare (Centering) is a C.E. model proven to improve health outcomes and reduce health disparities in other populations, such as pregnant women, people with diabetes, and sickle cell disease. However, no research exists to determine if Centering can be adapted to meet the unique HIV prevention needs of sex workers.

**Objective:**

We aim to explain the process by which we collaboratively and iteratively adapted Centering to meet the HIV prevention and PrEP retention needs of sex workers.

**Methods:**

We utilized the Assessment, Decision, Adaptation, Production, Topical Experts, Integration, Training, Testing (ADAPT-ITT) framework, a model for adapting evidence-based interventions. We applied phases one through six of the ADAPT-ITT framework (Assessment, Decision, Adaptation, Production, Topical Experts, Integration) to the design to address the distinct HIV prevention needs of sex workers in Chicago. Study outcomes corresponded to each phase of the ADAPT-ITT framework. Data used for adaptation emerged from collaborative stakeholder meetings, individual interviews (*n* = 36) and focus groups (*n* = 8) with current and former sex workers, and individual interviews with care providers (*n* = 8). In collaboration with our community advisory board, we used a collaborative and iterative analytical process to co-produce a culturally adapted 3-session facilitator's guide for the Centering Pre-exposure Prophylaxis (C-PrEP +) group healthcare model.

**Results:**

The ADAPT-ITT framework offered structure and facilitated this community-empowered innovative adaptation of Centering Healthcare. This process culminated with a facilitator's guide and associated materials ready for pilot testing.

**Conclusions:**

In direct alignment with community empowerment, we followed the ADAPT-ITT framework, phases 1–6, to iteratively adapt Centering Healthcare to suit the stated HIV Prevention and PrEP care needs of sex workers in Chicago. The study represents the first time Centering has been adapted to suit the HIV prevention and PrEP care needs of sex workers. Addressing a gap in HIV prevention care for sex workers, Centering PrEP harnesses the power of community as it is an iteratively adapted model that can be piloted and replicated regionally, nationally, and internationally.

**Supplementary Information:**

The online version contains supplementary material available at 10.1186/s12889-023-17508-4.

## Background

Sex workers, those who exchange sex for money or nonmonetary items [1], are disproportionally impacted by HIV [2]. Those engaging in sex work practice in a variety of settings, both indoors and outdoors. In the United States, sex workers are often a hidden, complex-to-access population [3, 4] and thus underrepresented in HIV prevention research [5]. Due to the criminalized nature of sex work, few individuals self-identify as sex workers, making quantifying the actual number of sex workers in Chicago challenging. Arrest data is often used as a proxy to quantify sex work, and between 70,000–80,000 sex work-related arrests occur annually. However, it is estimated that in the United States, there exist between one million and two million women engaging in sex work [6, 7].

The current legal framework in the U.S. is that sex work is illegal in all 50 states, minus a few specific jurisdictions within brothels in Nevada [8]. In Chicago, IL, sex work is not only criminalized, but prostitution-specific ordinances exist authorizing discretion to police officers about how to respond to someone believed to be engaging in sex work. Arrests, tickets, fines, and jail time are all potential outcomes for those Chicago police believe to be engaging in sex work. While Federally Qualified Health Centers exist throughout the city of Chicago, fear of criminalization, stigmatization, and discrimination serve as barriers to accessing care. Further, the hidden, illegal nature of sex work makes tailored HIV prevention efforts for sex workers challenging to implement. The same structural barriers to care access also contribute to a gap in HIV prevention research among sex workers because, until recently, sex workers have not been partners in research development [9, 10]. While it may be argued that sex workers are indeed an over-researched population, especially in HIV research, their needs and priorities have not been adequately addressed [2, 11–13]. Research is needed to facilitate the development of evidence-based interventions specifically tailored to their needs [5].

Community-empowered interventions, however, are evidence-based and have been a cornerstone for reducing HIV transmission among sex workers because community-empowered interventions are designed, implemented, and evaluated by the community served [9, 14]. Centering Healthcare (Centering) is a community-empowered intervention that has been successful with other populations experiencing health inequities. Aimed at addressing the healthcare, learning, and community-building needs of pregnant patients, Centering originated as a group model of prenatal care but has since been successfully adapted for other populations, including those with sickle cell disease, diabetes, and as a method of postpartum HIV prevention [15]. As such, Centering may be well-suited for HIV prevention among sex workers as it has excellent potential to fit within current health systems, meet the HIV prevention needs of sex workers, and increase the probability of sustainable care [9, 16–19]. Centering studies report positive outcomes, including increased condom use, fewer repeat pregnancies, lower pre-term birth risk, more knowledge and satisfaction, and more care visits [20–22].

### Why community empowered interventions?

Sex workers are particularly vulnerable to HIV/STIs due to increased exposure to trauma, structural violence, and social barriers. These complex factors, in addition to high rates of intimate partner violence, may reduce autonomy over health-promoting behaviors such as consistent condoms, thus increasing vulnerability to HIV/STI [1, 9, 23]. In addition, barriers in the healthcare setting have resulted in sex workers being less likely to receive comprehensive healthcare, STI screening, treatment, and HIV prevention services due to structural barriers [23]. Historically, those with increased vulnerability to HIV, such as sex workers, have been excluded from the process of intervention development, thereby limiting the effectiveness of interventions. Community empowerment approaches may help to overcome these limitations as it is a collective process "whereby sex workers are empowered and supported to address the structural constraints to health and improve their access to services to reduce the risk of acquiring HIV" [9], p.172]. Community-empowered interventions aim to decrease vulnerability to STIs, including HIV, social, and structural barriers while increasing individual, financial, and community resources and social support [24]. Therefore, community-empowered, innovative approaches to preventing HIV among sex workers are needed [9, 25].

### Why Centering Healthcare for PrEP care?

PrEP is a medication that protects against HIV infection [26]. For those currently uninfected but at an increased risk, taking a daily pill is an effective HIV prevention method. It is an excellent option for sex workers that requires no partner negotiation, is user-controlled, and is cost-effective [26, 27]. PrEP is less effective when not taken as prescribed. As such, innovative and sustainable ways to foster PrEP initiation and adherence among those with increased vulnerability to HIV remain a public health priority [1, 26, 27]. Adapting Centering to meet the PrEP care needs of sex workers in Chicago aims to bolster the efficiency of healthcare personnel while also enhancing the healthcare experience of the patients served. A preponderance of evidence suggests that Centering impacts outcomes for disadvantaged groups, including those from historically under-resourced communities struggling with sickle cell disease, diabetes, and interstitial cystitis, thereby highlighting its potential relevance for efficient and effective PrEP care for sex workers [15, 28, 29].

### Why ADAPT-ITT?

The Assessment, Decision, Adaptation, Production, Topical experts-integration, Training, and Testing (ADAPT-ITT) model has been successfully used to tailor evidence-based interventions to meet the specific needs of communities with increased vulnerability to HIV [30, 31]. The model includes eight phases, and, in alignment with community empowerment, leans on the guidance and leadership of community members and stakeholders to effectively adapt an existing model to meet a new community's HIV prevention needs. Rather than reinventing the wheel, through ADAPT-ITT, researchers can build on what has been previously proven effective, i.e., Centering Healthcare. Utilizing ADAPT-ITT for our study ensured community involvement from conception to dissemination. In this paper, we describe the use of the ADAPT-ITT model for adapting the Centering Healthcare intervention to meet the HIV prevention and PrEP needs of sex workers in Chicago [30, 32]. The Centering model has been successfully adapted and used around the world. In alignment with the Getting to Zero 2030 initiative, this community-empowered Centering adaptation, focusing on HIV prevention among sex workers, has excellent potential to engage sex workers in consistent PrEP care by elevating the HIV prevention and community-building needs of this marginalized population.

## Methods

We used the ADAPT-ITT framework to guide the adaptation process. Specific steps incorporated within each phase are described within the paper and summarized in Table 1. Formative research took place in six stages between January 2019 and March 2022. Our team included researchers with expertise in Centering Healthcare, intervention development, adaptation and implementation, Health Equity research, and a community advisory board that included current and former sex workers, healthcare providers, social workers, and representatives from an FQHC in Chicago. A diverse representation of 13 authors contributed to this work, including six white cisgender women, 2 Black cisgender women, 2 Black cisgender men, 1 Black nonbinary person, 1 Arab cisgender woman, and one white cisgender man.

**Table 1:** Applying the ADAPT-ITT model to guide the adaptation of centering healthcare

Phase	Methodology	Results or Observations
**1. Assessment**	• Conducted meetings with Community Stakeholders • Conducted elicitation interviews with Centering Healthcare experts • Formative evaluations with FQHC • Formed a Community Advisory Board • Conducted individual with (*n* = 36) sex workers in Chicago	• The priority population, sex workers in Chicago, requires interventions that are community empowered and specific to their unique needs • The intervention needs to address HIV prevention, Pre-Exposure Prophylaxis (PrEP), and sexual health promotion • Themes and sub-themes from all qualitative interviews were developed; PrEP stood out as a major theme • A Community Advisory Board (CAB) facilitated by a former sex worker to promote community empowerment was created • CAB members were selected for the study, met over Zoom and provided regular feedback on the proposed activities and plan • CAB facilitator and CAB members were paid for their time and consultation • The CAB and community stakeholders determined a HIV PrEP prevention navigation program was adequate to suit the stated needs of the sex worker community
**2. Decision** (What Evidence-Based Intervention will be used or adapted?)	• Decided to adapt Centering Healthcare model to suit the HIV prevention needs of sex workers in Chicago	• Centering Healthcare (Centering), a multi-session evidence-based intervention was selected because this model of group care has been successful with health inequity populations receiving regular prenatal, diabetes, and sickle cell care • This group healthcare model was selected in response to a mutual desire for interventions that promote healthcare, education and community building • Centering Healthcare has never been adapted to suit the HIV prevention needs of sex workers, but the health assessment, interactive learning, and community building core elements align with community empowered health interventions • The decision was made to adapt three Centering Healthcare sessions to suit the needs of PrEP-naïve sex workers in Chicago desiring PrEP for HIV prevention
**3. Adaptation** (Methods used for adaptation)	• Conducted virtual CAB meetings where general overview of Centering was presented • Themes from Phase 1 interviews were used to guide adaptation • Retained core elements of Centering model: Health Assessment, Interactive Learning, Community Building • Adaptation would involve the materials used, and the various activities within modules of EBI	• CAB members engaged in each meeting session and were excited about adaptation process • CAB members reported experience of adaptation as healing and inclusive • CAB members expressed concerns, suggested changes, and omission of some of activities, specifically the dietary intake activity • CAB members expressed interest in an activity to dispel myths about HIV and PrEP • HIV PrEP: “Word on the Street” activity replaced dietary intake • Scheduled additional CAB meetings to thoroughly present and review each session and each activity within each session
**4. Production** (How is the first draft produced and how are modifications to the original EBI incorporated?)	• Designed three 2-h Centering PrEP (C-PrEP +) sessions • Centering experts, interventionists and community liaisons incorporated themes from individual interviews and CAB feedback to inform a draft of three C-PrEP + sessions by discussing various potential activities within each module • Selected FQHC for formative pilot testing • Developed activities for all three sessions • First draft of facilitator’s guide developed	• Developed the first draft of three sessions of an HIV prevention model of Centering for sex workers: Centering Around PrEP (C-PrEP +) • Centering core elements were facilitated by having Centering experts present for adaptation process • Centering core elements were maintained utilizing this iterative group process •Community empowerment maintained by having former sex worker facilitate the community advisory board adaptive process
**5. Topic Experts** (Who supports adaptation process?)	• Initial draft of C-PrEP + was reviewed by topical experts and adapted collaboratively with CAB • Centering Healthcare experts reviewed model adaptation to ensure consistency of C-PrEP + with the Centering Healthcare model Four CAB meetings allowed for stakeholder feedback on the content, materials and acceptability of C-PrEP + ; this feedback was synthesized and incorporated to inform a second draft of the model and associated facilitator’s guide	• CAB members voiced need for meditative/reflective component, which was then added to each session • CAB members suggested opportunities for anonymous questions; recommendation incorporated in the form of an anonymous question basket • Centering healthcare experts expressed that C-PrEP + adaptation maintained the core components of the Centering Healthcare model: health assessment, interactive learning and community building • Draft 2 of facilitator’s guide and associated materials completed
**6. Integration** (What components will be incorporated into the adapted EBI?)	• Focus groups were conducted with sex workers and healthcare providers to elicit input on the adapted activities • Care providers furnished advice regarding HIV prevention care • Collaborating sex workers offered advice regarding appropriate fit of activities	• Care providers expressed concern about potential offensive and exclusive use of casual language in relation to gender identity and body parts • Sex workers expressed appreciation for casual language and light-hearted interactive games, specifically related to gender identity and body parts • Sex workers expressed discomfort with the role-playing activity. While they believed that discussing safety and negotiation was necessary, the idea of a role play was reported as seeming too vulnerable for sex workers • The role play scenario activity was replaced with Mental Checklist for Safety Activity • Revised meditation activity language as suggested by care providers • Adapted the C-PrEP + model to elevate the needs and recommendations of sex workers while incorporating input from care providers and centering experts • Draft 3 of facilitator’s guide an..d associated materials completed
**7. Training**	In process	• Trainings with Centering Healthcare have been scheduled for healthcare provider and peer facilitators • Trainings will be complete by September 2023
**8. Testing**	Preparing for testing	• IRB submitted for approval to begin pilot testing; awaiting approval • Received funding to support pilot testing; NINR

Additionally, six contributing authors identify as LGBQ + , and socioeconomic class spanned from working class to middle or upper middle class. Regarding potential biases, we recognized how our own experiences of racism, sexism, ageism, and the intersection of these identities may influence how we understand and interpret participants' experiences [33]. Therefore, we were careful not to make assumptions or draw conclusions about participants' experiences from prior work or based on our own experiences; however, as a few co-authors shared identities with the participants, we think our lived experiences strengthened the research process. To protect the privacy of additional authors, we will not disclose who is a current or former sex worker. However, it is essential to note that current and former sex workers were involved in every stage of the process. They served in leadership roles by running CAB meetings, co-developing the semi-structured interview guide, conducting interviews, and collaboratively analyzing the data and disseminating findings.

### Recruitment

Participant recruitment focused primarily on adults engaged in sex work. For this study, sex workers are considered those who exchange sex for money or nonmonetary items [1]. Sex worker participants were considered eligible if they were over 18, traded sex for money or nonmonetary items within the last 12 months, spoke English, and were willing to participate in audio-recorded individual or focus group interviews addressing their HIV prevention and sexual health self–management practices. Later in the study, care providers were recruited to participate in focus groups. At various stages of the study, participants were either passively recruited through clinic-based flyers, social media (i.e., Twitter, Facebook, Instagram), and private community list-serves or actively recruited via word-of-mouth referrals. Whether a care provider or a sex worker, all potential participants emailed or called the study team to assess eligibility and learn more about the study; if eligible and interested, a remote individual or focus group visit was scheduled. Recruitment was done virtually, and interviews were done over Zoom due to the restriction of in-person events due to COVID-19 precautions. While we could not provide internet access to individual participants, some participants could access broadband through various networks, including public outside spaces where internet usage was free and accessible.

### Ethical considerations

The institutional review board of University of Illinois Chicago and the FQHC approved all study procedures. Conducting research with participants who engage in sex work has unique ethical considerations, as this is a population that experiences criminalization and targeted policing. Facilitating confidentiality within individual interviews and focus group sessions was essential to protect those who trade sex and those who have multiple marginalized identities in addition to being a sex worker.

- The Zoom sessions, individual interviews, and focus group interviews were audio recorded. Participants were assigned a unique code number used only for this study on a digitally recorded file and transcript to protect participant anonymity. In addition, many participants chose not to turn their cameras on. De-identified audio recordings were transcribed by a professional transcription service, with incidental identifiers removed during transcription. Individual interview data were coded, stored on a password-protected computer, and encrypted to prevent access by unauthorized personnel; any identifiers, raw audio recordings, and contact information were destroyed after data collection.
- Focus group sessions provide opportunities for conversation and shared insight; however, they are less confidential than an individual interview. While focus group participants were encouraged to maintain confidentiality, confidentiality could not be assured post-focus group. To further minimize these risks, the researchers asked all focus group members to respect each other's privacy and confidentiality and not identify anyone in the group or repeat what was said during the group discussion.

### Phase 1 (Assessment)

We conducted a community health assessment and regular discussions between the research team and the community partners, where we identified the need to develop an HIV prevention and PrEP navigation program.

### Procedures

We held quarterly two-hour advisory board meetings with the FQHC leadership and FQHC-affiliated healthcare providers. We formed a 10-person Community Advisory Board (CAB) that was co-facilitated by a former sex worker and a respected community member and comprised of current and former sex workers, outreach workers, caseworkers, healthcare providers, researchers, and Centering experts. In addition, we conducted elicitation interviews (*n* = 6) with Centering Healthcare experts to gain insight into this model of care [34]. To assess that care providers, staff, and building space would be adequate to suit the Centering project and to meet the cultural safety needs of the community, we conducted formative evaluations of the FQHC's existing resources. We also conducted one-on-one interviews and collected demographic surveys to assess the knowledge, attitudes, beliefs, perceived risk, barriers and social norms, self-efficacy, and intentions related to HIV prevention, HIV self-management, and HIV harm reduction with sex workers (*n* = 36) in Chicago [14, 35, 36].

The inclusion criteria for the individual interviews with sex workers included the following: a) age 18 or older; b) exchanged oral, vaginal, or anal sex for something of value in the past 12 months; c) live in the Chicago area; d) speak and understand English; and e) be willing and able to provide informed consent to participate in an individual interview. Rapid content analysis was used to identify themes from all qualitative interviews, where PrEP emerged as a significant theme. All the sex workers interviewed were asked about PrEP, and many described both interest in and barriers to taking PrEP to prevent HIV. One participant stated, "*Hey, if I'm participating in sex work and I have not contracted HIV, then I can take Prep, and it can help protect me against it… when you take that information and pass it around… it's helping someone*" (36-year-old, Black, cisgender male). When asked about interest in PrEP, another participant acknowledged her challenge around accessing PrEP, stating, "*I feel like it (PrEP) was inaccessible… but yeah, I think, ideally, I might use it*" (47-year-old Latinx cisgender woman). In line with individual interview findings, focus groups and CAB members acknowledged the need for PrEP care to be community-empowered and accessible. Other healthcare-related themes (See Table 2.) included seeking unbiased patient-centered care, peer-involved care, and community-building opportunities [14, 36]. While initial CAB meetings were held in person, data collected after March 2020, including the individual interviews, occurred over Zoom due to the COVID-19 pandemic restrictions, limiting participation to those with internet access.

**Table 2:** How quote-driven themes align with the first draft of Centering PrEP (C-PrEP +)

Themes	Quotes from Sex workers	Lessons Learned	Alignment with Centering
PrEP:	*If I wasn't taking it (PrEP), I would say the risk was probably high … I have multiple partners. I’m polyamorous. I'm a slut, professionally and personally. I like to have fun and do my things and, I'm safe* (28-year-old, white, gender queer)	PrEP was seen as effective HIV prevention affording people to live the life that they intend to live, while also remaining safe	Centering Healthcare aims to enhance preventative practices by facilitating self-management, personal empowerment, and bodily autonomy
Unbiased-Patient-Centered Care	*They just do a really good job of not judging. They're used to seeing people of so many different kinds of backgrounds and experiences. They do a lot of work to de-stigmatize HIV and infection, being trans, and also de-stigmatize sex work. I feel very welcomed in that space, because I feel like there's a lot of people there whose identities and experiences in some ways intersect with mine. And I don't feel that way when I go to other health settings.* (31-year-old, Black, cisgender woman)	Sex workers expressed feeling most safe with healthcare experiences when the care providers and staff were reflective of their own identities. The experience of congruous care was described as unbiased, and patient centered	Centering Healthcare aims to center patient needs with intentionality, comfort, continuity, and peer-to-peer engagement
Peer-Involved Care	*I disclosed being a sex worker only one time when I got tested for HIV. It's because the person testing me was once a provider (sex worker) themselves … I felt just comfortable enough because they were also part of the industry. On top of that, they were considerate of me. They understood. They had empathy.* (30-year-old, white, transgender woman)	Sex workers expressed preferring to be in spaces with others who identify as a sex worker or as queer	At the heart of Centering is peer-to-peer interaction, which bolsters engagement in care. Also, having peer co-facilitators fosters trust in the care delivery model because peer facilitators can connect about shared experiences that care providers without lived experience lack
Community Building	*Like a community of people who want to talk about what your healthy sexual life looks like, and I feel like if I just had a piece of that, I don't know if it would feel as hard as it does.* (27-year-old, mixed-race, cisgender woman)	Sex workers expressed desiring consistent opportunities to build community related to their health and well-being	Community building is a strength of Centering Healthcare and is successful because the community becomes more cohesive with subsequent visits. The same patients and facilitators foster dependable opportunities for community building

### Phase 2 (Decision)

The CAB determined that Centering would be adapted from its original purpose as a group prenatal care model to meet the HIV prevention and PrEP care needs of sex workers in Chicago. The CAB's decision was based on the evaluation of the information gathered in phase 1 (Assessment) and previous evidence of the effectiveness of the Centering model [20, 21, 29].

### The rationale for the choice of Centering

Centering is an evidence-based community-empowered model with demonstrated effectiveness for reducing health inequities in various patient populations and disease types [20, 21, 29]. It has since been modified and adapted to address the needs of diverse patient populations. Rather than a one-on-one visit, a cohort (or group) of 8–12 patients meet with the same providers at each visit for regular health assessments, linkages to services, and 75–90 min of interactive learning and skill-building that centers on patients' experiences. The Centering Healthcare model emphasizes social support and joint problem-solving through Health Assessment, Interactive Learning, and Community Building [21].

Centering has been shown to improve individual health through group engagement. The group process enhances learning, promotes healthy behavior change, builds a sense of control over health while developing a supportive network, and creates a collaborative provider–client relationship through continuity of care (Baldwin, 2006; Klima et al., 2009). Another strength is Centering's clear guidelines, which allow for program replication. Participants had a favorable response to the Centering Model. For example, when asked about adapting Centering to meet the PrEP care needs of sex workers, one participant acknowledged the benefit of peer support by saying, "*Yeah, I think that'll be super helpful in engaging, to hear about tips that other people have…because it's really useful for people to kind of share and come together. If we're thinking about Centering, this is not an opportunity that maybe they have, especially if they don't know a lot of people who are exchanging sex or in sex work. It could be nice to have this outlet and just kind of engagement*" (40-year-old, Black, transgender woman).

### Phase 3 (Adaptation)

University researchers and community stakeholders collaborated with the FQHC to conceptualize how to adapt Centering for sex workers initiating PrEP.

### Procedures

We conducted virtual sessions with the existing CAB, during which we reported the findings from the individual interviews conducted during the assessment phase. We discussed barriers and facilitators to accessing healthcare during and before the pandemic. We also presented a general overview of Centering and various activities implemented in different sessions. We then discussed how Centering could be adapted to address PrEP care and health promotion needs of sex workers in Chicago. CAB members agreed to retain the three core elements of the Centering model (Health assessment, Interactive Learning, and Community Building). Still, they suggested adapting certain activities to be responsive to sex worker culture regarding HIV prevention. For example, CAB members recommended revising language about body parts or creating opportunities to use humor for icebreakers. For more examples, refer to Tables 2 and 3.

**Table 3:** How C-PrEP + maintains fidelity to the Centering Healthcare model

Essential Elements of Centering	Suggested Activities	Example
Health Assessment	• Self-Assessment • Care Provider Assessment	• Self-Swab for GC/CT • Urinate in cup to assess for PrEP • Self-Assessment single question • Meet briefly with care provider in private area away from the circle
Interactive Education	• Skill building • Each session has a plan • Emphasize response to group needs • Group guidelines established and reiterated	• Question Basket • Word on the Street • Facilitator’s guide used to conduct session plans • Facilitators guide rather than control activities in each session • Facilitators include one trained peer
Community Building	• Meditation • Group guidelines • Socializing • Group size	• Circle to facilitate sharing • Grounding stone • Artful expression • Sharing intentions

### Phase 4 (Production)

This phase included adapting activities and materials needed for the targeted intervention in the three sessions.

### Procedures

Our team of Centering experts, interventionists, and community liaisons incorporated themes from individual interviews and CAB feedback to develop the first draft of three 2-h sessions of an HIV prevention model of Centering for sex workers, which we named Centering PrEP (C-PrEP +). All three sessions of the C-PrEP + model include health assessments, interactive learning, and community building. Each session focuses on a theme and has corresponding activities. The theme for session one is an orientation to Centering & mindfulness. Discussion topics include C-PrEP +, PrEP-related knowledge, and health management practices. For example, a session one activity is called "HIV & PrEP: Word on the Street." The goal of this activity is to dispel myths about HIV and PrEP. Session two focuses on two topics: COVID-19 and coping mechanisms. Discussion topics include barriers to adequate health care, side effects of PrEP, substance use, individual and community impact of COVID-19, and identification of effective coping strategies. The theme for session three is harm reduction behaviors. This session aims to identify sexual behaviors that stand in the way of safety while working and improving behaviors to decrease HIV risk.

Some topics and activities will facilitate effective communication, negotiation, and safe sex practices. The corresponding activity for safe sex practices in the C-PrEP + model is called "Mental Checklist for Safety." Facilitators will guide participants to create a short, memorable checklist they can refer to when engaging in sex work. For more details about C-PrEP + content, see Appendix 1 and 2. Through an iterative process facilitated by a former sex worker, we developed the first draft of C-PrEP + and the facilitator's guide. We adapted and created all the sessions' activities while maintaining Centering's core elements. We also finalized the selection of the collaborating FQHC as the site for formative pilot testing.

### Phase 5 (Topical Experts)

The topical expert phase leans heavily on those with proficiency in the content being adapted (HIV prevention and PrEP care), know-how related to model adaptation (Centering), and knowledge about the population to be served by the model (sex workers). Topical experts are distinct from the CAB members and are asked to give feedback on the adaptation process and the materials used. Topical experts included two former sex workers, one centering healthcare interventionist, one social worker, one legal expert, and two sexual health FQHC clinicians. The topical expert phase presented the first draft of the C-PrEP + model to the identified experts described below. We then collected feedback to adapt this community-empowered model further. After modifying the model according to expert feedback, we then conducted focus groups with sex workers and care providers together to elicit targeted feedback.

### Procedures

We developed quality assurance and process measures by identifying topical experts in HIV prevention, sex work, and Centering Healthcare. Distinct from study participants and community advisory board members, topical experts included sex workers as well as stakeholders from HIV-focused community organizations, public health experts, and centering experts. We collected feedback on the content, materials, and acceptability of C-PrEP + . For example, topical experts suggested incorporating meditation/reflective components into each session and providing opportunities for anonymous questions during the activities (see Table 3). The corresponding facilitator's guide and C-PrEP + model activities were adjusted based on the topical expert's feedback. Additional feedback elicited through semi-structured focus group meetings with sex workers and care providers further informed the C-PrEP + adaptation.

### Focus group sessions with sex workers and care providers

Traditional ADAPT-ITT theater testing allows study participants (in this case, sex workers and care providers) to experience and respond to the proposed intervention before pilot testing. Due to COVID-19 restrictions, we were unable to conduct traditional theater testing. Instead, we conducted four focus group sessions where sex workers and care providers together were introduced to Centering content and corresponding activities to extract input on the adapted activities (see Tables 3 and 4). Sex workers provided feedback on activities and offered suggestions for targeted adaptation, while care providers offered recommendations related to PrEP care protocol and patient engagement. At the end of this phase, we developed the second draft of the model and the corresponding facilitator's guide.

**Table 4:** How focus groups informed activity adaptations

Original Activity	FG Recommendations	Adaptation
Role-playing regarding sexual coercion	Safety, health promotion, disease prevention more appropriate use of activity time	Added mental checklist for personal safety
Session opens with participants in a circle and a deep breathing exercise	Verbally reaffirm and reiterate confidentiality and trust at the start of each session	Added verbal reaffirming confidentiality statement to the original activity

### Participants

The inclusion criteria for sex workers to participate in the focus group sessions included: a.) age 18 or older; b.) exchanged oral, vaginal, or anal sex for money or nonmonetary items in the past 12 months; c.) live in the Chicago area; d.) speak and understand English; and e.) consent to actively participate in an audio-recorded, two-hour focus group session. The inclusion criteria for providers to participate in the focus group sessions included: a) be an R.N., APRN, or physician who cares for those engaged in sex work; b) speak and understand English; and c) consent to actively participate in an audio-recorded, two-hour focus group session. Qualitative results from the focus groups highlight how input from participants contributed to model adaptation. For example, a 32-year-old Black female sex worker acknowledged how collaborative transformation made the model more relevant. *"I just want to say that I really appreciate the iterative process of the work that you're doing. The going to the community, getting information, coming together, creating something, bringing it back to community, gain feedback, sort of over and over. I really appreciate that process…. Y'all hitting what's trending now*." Tables 3 and 4 expand on how focus group sessions informed the model adaptation.

### Phase 6 (Integration)

Feedback from topical experts and findings from the sex worker and care provider focus groups informed the third draft of the model adaptation. We reviewed and adapted the second draft of C-PrEP + collaboratively with CAB members based on the analysis of the focus group sessions and integrated the findings to develop the third draft of the model and associated facilitator's guide. The following section describes the final adaptations made to the original activities.

### Adaptations made to original activities

As shown in Table 4, focus group and CAB feedback impacted how C-PrEP + was adapted. Role-plays that focused on condom negotiation were included in the initial two drafts of the C-PrEP + facilitator's guide and associated materials. Role-playing, the experience of negotiating with sex work clients, was an adaptation from the original Centering model, which utilizes opportunities for role-plays to facilitate interactive learning and skill-building. Role-plays around sexual coercion and associated negotiation were deemed culturally unsafe by topical experts and were ultimately removed. Instead, CAB members and topical experts did agree that providing opportunities to discuss circumstances that impact safety would be necessary for the health promotion and disease prevention of sex workers. Therefore, CAB and topical experts determined an activity called "Mental Checklist for Personal Safety" to be a culturally safe alternative to role-plays. This activity utilizes four different tables (each with a different theme, such as Communication and Negotiation, Physical Items and Mental Preparation, Safety, and Other-Participants' Choice). It aims to develop a personal checklist of four items/ideas to promote health and reduce harm when partaking in sexual activities. Here, participants would be separated into three groups. Each group would rotate to different tables where they could add their ideas to a sheet of paper, creating a brainstormed list. Participants will return to the circle once each group has rotated to each table. Each participant will be given a notepad and pen to write out their mental checklist during the discussion. Facilitators will start the conversation by acknowledging the items listed at each table (for example, possible items and ideas listed include communicating about condom usage, taking PrEP medication if applicable, having PPE and travel sanitizer on hand, and being prepared by learning self-defense or carrying pepper spray).

Another adaptation that C-PrEP + takes from the original Centering model is the style for starting each session. The Centering model opens sessions in the community and a circle, sometimes with three deep breaths and a chime. The CAB and topical experts agreed that deep breathing and a calming sound are practical tools, but there should also be a verbal opening about privacy and trust. Therefore, to align with culturally safe and community-empowered practices, participants will collaboratively create a mission statement about group privacy, trust, and confidentiality, which will be reaffirmed at the beginning of each session.

Based on study results and CAB input, new activities were added to Centering the development of C-PrEP + , which maintains the model's fidelity. Still, themes are responsive to the stated needs of the community (Appendix 2). The first of these themes surrounded the discussion of gender identity and body part naming. This diverse population of individuals utilize their bodies in the work they do and includes a significant number of transgender, gender fluid, gender non-conforming, and gender expansive individuals. To avoid triggering and stigmatizing language when discussing body parts, topical experts and the CAB agreed that it would be essential to have an activity related explicitly to body parts and genitalia naming. The aim will be to create a respectful environment for discussions about the physical body to ensure that the language is appropriate and respectful. The CAB members developed a genital name game that sex worker topic experts agreed was a fun and light way to address the need for respectful language. During this activity, participants will be asked to list (on a whiteboard) the names used to describe private parts. After as many names as possible are written on the board, the group will be asked to discuss names that could be triggering to the participants. While CAB members and self-identified sex workers believed that categorizing private parts names as "thumbs up" and "thumbs down" was fun, straight to the point, and engaging, healthcare provider topical experts were concerned that the thumbs up or thumbs down could be felt as stigmatizing or ostracizing. Responsively, both CAB and all topic experts agreed to remove the thumbs-up or thumbs-down component and determined that a structured discussion about agreed-upon private part names would be an essential activity to promote cultural safety within a group setting.

To close the sessions, the researchers, CAB, and topical experts aimed to be responsive to participants' desire for spiritual and meditative components as part of their care. For this reason, we have added a grounding stone for personal or shared intentions. This activity is intended to provide solidarity at the end of every session. The group will come together, hold a stone supplied by the research team, and say or think a positive affirmation or meditate with a positive intention for someone else to pick up and receive. These stones will touch and blend. Each participant will be invited to carry a stone throughout the week to symbolize positivity, empowerment, and solidarity. Participants will have three stones at the end of the three C-PrEP + sessions.

## Discussion

This study filled a critical gap in Centering adaptation and in community-empowered research with sex workers considering Prep. While Centering has been successfully adapted, the model has never been iteratively adapted with community members and stakeholders, nor has it been adapted to meet the PrEP care needs of sex workers. The ADAPT-ITT framework offered structure in adapting the Centering model to suit the stated requirements of sex workers and care providers. Key insights, including spirituality and meditation elements, resulted in specific adaptions, such as using a grounding stone to emphasize intentionality. To address HIV disparities among sex workers, we directly addressed structural barriers to care like stigma and healthcare discrimination by incorporating a community-empowered intervention, C-PrEP + , that engaged sex workers throughout the intervention development process [25, 37]. We developed a C-PrEP + facilitator's guide outlining facilitator expectations and detailing the goals and objectives for each of the three C-PrEP + sessions. This program has great potential for improving PrEP adherence, which has been shown to prevent 99% of sexually transmitted HIV infections [27]. To empower marginalized sex workers to have autonomy over their health in complex and often violent work environments, C-PrEP + has excellent potential to fit within current health systems, meet the HIV prevention needs of sex workers, and increase the probability of sustainable care [9, 16–19]. Such an intervention integrates support that will, directly and indirectly, impact HIV/STI prevention, allowing the potential for sex workers to be empowered, see reductions in HIV/STI transmission, and improve other aspects of their health.

### Limitations

The ADAPT-ITT framework has been proven valuable, and many studies have successfully employed this scientific framework; however, due to COVID-related challenges, the model was only partially adopted in the current study. Future studies should include all phases of the model. We have received funding to complete the training and testing phases. As this research was conducted during the COVID-19 pandemic, all study procedures were conducted over Zoom, limiting participation to those with internet access. This need for internet access may have resulted in a more stable, less structurally vulnerable population, which may not represent the broader Chicago SW community. Due to COVID-19 restrictions on in-person research, traditional theater testing was not implemented. Instead of theater testing, we presented the model by describing each session and each activity over Zoom through regular stakeholder meetings. These meetings allowed us to receive feedback about the model adaptation. Although the sample size of this study is small, the study findings show the reflection of diverse experiences among sex workers during the pandemic. However, input received from various stakeholder groups should be noted as a strength of the study. Future work should consider larger sample sizes and in-person theater testing to replicate the findings of this study.

Another limitation is that we have yet to pilot this adapted model. The following steps involve the final two phases of the study: training and testing. With funding from the National Institute for Nursing Research, we have plans to pilot this culturally adapted Centering model (C-PrEP +) at one FQHC to determine the feasibility and acceptability of addressing the HIV prevention and PrEP care needs of sex workers in Chicago. This piloting includes step-by-step training for C-PrEP + facilitators and lunch-and-learn activities for those who can recommend this group care model. Using an implementation science framework, we aim to continue our partnership with community members, Centering Health experts, researchers, and FQHC stakeholders to integrate C-PrEP + into the healthcare system. Despite the effectiveness of Centering [20, 21], no prior studies have evaluated whether it is a feasible and acceptable model among sex workers for HIV prevention and PrEP care.

## Conclusion

The primary goal of this study was to utilize the ADAPT-ITT model phases 1–6 to adapt Centering for sex workers desiring Pre-Exposure Prophylaxis (PrEP) for HIV prevention. Centering PrEP (C-PrEP +), a group model of PrEP care, was iteratively developed to provide an alternative to usual PrEP care for sex workers to increase community and individual empowerment and facilitate PrEP adherence through community support and skill-building. The iterative adaptive process to create the C-PrEP + model highlights the importance of implementing community empowerment approaches to improve health outcomes for sex workers. In addition to sex worker team leadership, scholars from numerous research institutions collaborated with a federally qualified health center (FQHC), community organizations, a community advisory board, and other stakeholders to ensure that the adaptation process was appropriately addressing the PrEP care needs of sex workers in Chicago. A tailored HIV prevention intervention, C-PrEP + aims to reduce HIV disparities among sex workers by focusing on community-empowered health promotion and PrEP adherence.

The adaptation process was overwhelmingly well-received by community members. CAB meetings often ended with members offering unsolicited gratitude for the consistent and participatory engagement. The ADAPT-ITT provides a framework for ensuring community engagement throughout the adaptation process [30, 31]. We also know that community-empowered interventions have been the most successful at preventing HIV among sex workers [24]. Though Centering has been adapted to meet the needs of other populations [15, 28, 29], this model has never been adapted utilizing the ADAPT-ITT framework, nor has it been adapted to suit the HIV prevention and PrEP care needs of sex workers. Centering PrEP addresses a gap in HIV prevention care for sex workers by harnessing the power of the community and by developing a model that can be piloted and then replicated regionally, nationally, and globally.

This research highlights a critical approach to intervention development among one highly marginalized population. Working in partnership throughout the research process, from conception through dissemination, elevates community members' voices. The community members who participated in the model adaptation are excited about the launch of this program. We are hopeful that piloting will be successful, given those who informed the model are the same people researchers aim to serve.

### Supplementary Information

**Additional file 1: Appendix 1. **Session Outline.

**Additional file 2: Appendix 2. **How C-PrEP+ Maintains fidelity to the Centering Healthcare Model.

## Data Availability

The datasets used and/or analyzed during the current study are available from the corresponding author upon reasonable request.
