# Supplementary material for: Centering PrEP: Utilizing ADAPT-ITT to inform group PrEP care for sex workers in Chicago
Source: BMC Public Health. 2024 Jan 2;24:56. doi: 10.1186/s12889-023-17508-4 (PMC10762989; doi:10.1186/s12889-023-17508-4)
Supplement: Supplementary file 1 — Additional file 1: Appendix 1. Session Outline. [file 12889_2023_17508_MOESM1_ESM.docx]

**Appendix 1: Session Outline**

| **Session** | **Health Assessments** | **Topics** | **Corresponding Activities** |
| --- | --- | --- | --- |
| **Session 1**  (1 month after rapid start)  Centering Orientation & Mindfulness | - Self-report, PrEP adherence and urine assay for self-monitoring purposes - HIV rapid test - STI self-screen - 1:1 with provider | - Welcome to C-PrEP+ - PrEP knowledge - Self-management | - Orientation, Group Guidelines - Building: HIV & PrEP “Word on the Street” - Genital Names |
| **Session 2**  (3 months after rapid start)  COVID-19 and Coping Mechanisms | - Self-report, PrEP adherence and urine assay for self-monitoring purposes - HIV rapid test - STI self-screen - 1:1 with provider | - Barriers to care - PrEP side effects - COVID-19 *(May substitute another public health concern such as lack of abortion access or monkeypox)* - Substance Use | - How do I cope? |
| **Session 3**  (6 months after rapid start)  Harm Reduction | - Self-report, PrEP adherence and urine assay   for self-monitoring purposes   - HIV rapid test - STI self-screen - Complete Metabolic Panel   1:1 with provider | - Communication and Negotiation - Safer Sex | - Art Collage - Mental Checklist for Safety |
