# Supplementary material for: Centering PrEP: Utilizing ADAPT-ITT to inform group PrEP care for sex workers in Chicago
Source: BMC Public Health. 2024 Jan 2;24:56. doi: 10.1186/s12889-023-17508-4 (PMC10762989; doi:10.1186/s12889-023-17508-4)
Supplement: Supplementary file 2 — Additional file 2: Appendix 2. How C-PrEP+ Maintains fidelity to the Centering Healthcare Model. [file 12889_2023_17508_MOESM2_ESM.docx]

**Appendix 2: How C-PrEP+ Maintains fidelity to the Centering Healthcare Model**

| **Essential Elements of Centering** | **Suggested Activities** | **Example** |
| --- | --- | --- |
| **Health Assessment** | - Self-Assessment - Care Provider Assessment | - Self-Swab for GC/CT - Urinate in cup to assess for PrEP - Self-Assessment single question - Meet briefly with care provider in private area away from the circle |
| **Interactive Education** | - Skill building - Each session has a plan - Emphasize response to group needs - Group guidelines established and reiterated | - Question Basket - Word on the Street - Facilitator’s guide used to conduct session plans - Facilitators guide rather than control activities in each session - Facilitators include one trained peer |
| **Community Building** | - Meditation - Group guidelines - Socializing - Group size | - Circle to facilitate sharing - Grounding stone - Artful expression - Sharing intentions for PrEP adherence / harm reduction |
